# Thymic rebound hyperplasia post-chemotherapy mistaken as disease progression in a patient with lymphoma involving mediastinum: a case report and reflection

**DOI:** 10.1186/s12893-021-01048-y

**Published:** 2021-01-14

**Authors:** Lei Qiu, Yi Zhao, Yang Yang, He Huang, Zhen Cai, Jingsong He

**Affiliations:** 1 Hematology & Tumor Chemotherapy Center, Zhoushan Hospital of Zhejiang Province, School of Medicine, Zhejiang University, No. 739 DingShen Road, Lincheng, New District, Zhoushan, China; 2 Bone Marrow Transplantation Center, The First Afliated Hospital, School of Medicine, Zhejiang University, No. 79, Qingchun Road, Hangzhou, China

**Keywords:** Thymic hyperplasia, Lymphoma, Recent thymic emigrants, Misdiagnosis, Case report

## Abstract

**Background:**

Chemotherapy can cause thymic atrophy and reduce T-cell output in cancer patients. However, the thymus in young adult patients has regenerative potential after chemotherapy, manifesting as thymic hyperplasia which can be easily mistaken as residual disease or recurrence in patients suffering lymphoma.

**Case presentation:**

This study reports a case of lymphoma in a young female adult who was initially diagnosed with an anterior mediastinal mass, and was found to have soft tissue occupying the anterior mediastinum repeatedly after chemotherapy, suggesting a lymphoma residue or disease progression. From discussions by a multi-disciplinary team (MDT), the anterior mediastinal mass of the patient was considered unknown and might be thymus tissue or tumor tissue, and it was eventually identified as thymus tissue via histopathology.

**Conclusions:**

The anterior mediastinal mass appearing after chemotherapy in patients with lymphoma can be considered as enlarged thymus, and such phenomenon is frequent in young adult patients who undergo chemotherapy or autologous hematopoietic stem cell transplantation. Additionally, detection of thymic output cells in peripheral blood might be a feasible approach to differentiate thymic hyperplasia from lymphoma.

## Background

The thymus, locating in the anterior superior mediastinum, is an important lymphoid organ as well as a major source of T lymphocytes which mainly produces almost-mature T lymphocytes and naïve T lymphocytes. It is established that the volume and function of the thymus vary with age. In individuals over 40 years, most thymus tissue is occupied by fat yet new T lymphocytes can still be produced at low levels [1, 2]. In addition to age, other factors like stress, toxins and viruses can also make an effect on the thymus. Chemotherapy, particularly, for malignant tumors, can make the thymus issue shrunken, leading to reduced production of naïve T lymphocytes and affecting cellular immunity function [1, 3–5]. However, with the elimination of these factors, thymic atrophy can recover, potentially resulting in a hyperplastic process known as thymic rebound hyperplasia (TRH) [5–7] which manifests as increased thymic volume and may be accompanied with recovery of thymic functions or even thymic hyperfunction. Lymphoma is one of the most common hematologic malignancies, with the thymus as a common invasion site. Hence, thymus tissue hyperplasia after chemotherapy is often mistaken as tumor recurrence and progression, especially in patients with lesions in the mediastinum. Here, this report presents a case of diffuse large B-cell lymphoma (DLBCL) with mediastinal lesions in which post-treatment thymic hyperplasia was mistaken as an event of disease relapse.

## Case presentation

A young lady aged 28 years old was admitted to our hospital for a cervical mass on September 20, 2016.

**Fig. 1:**
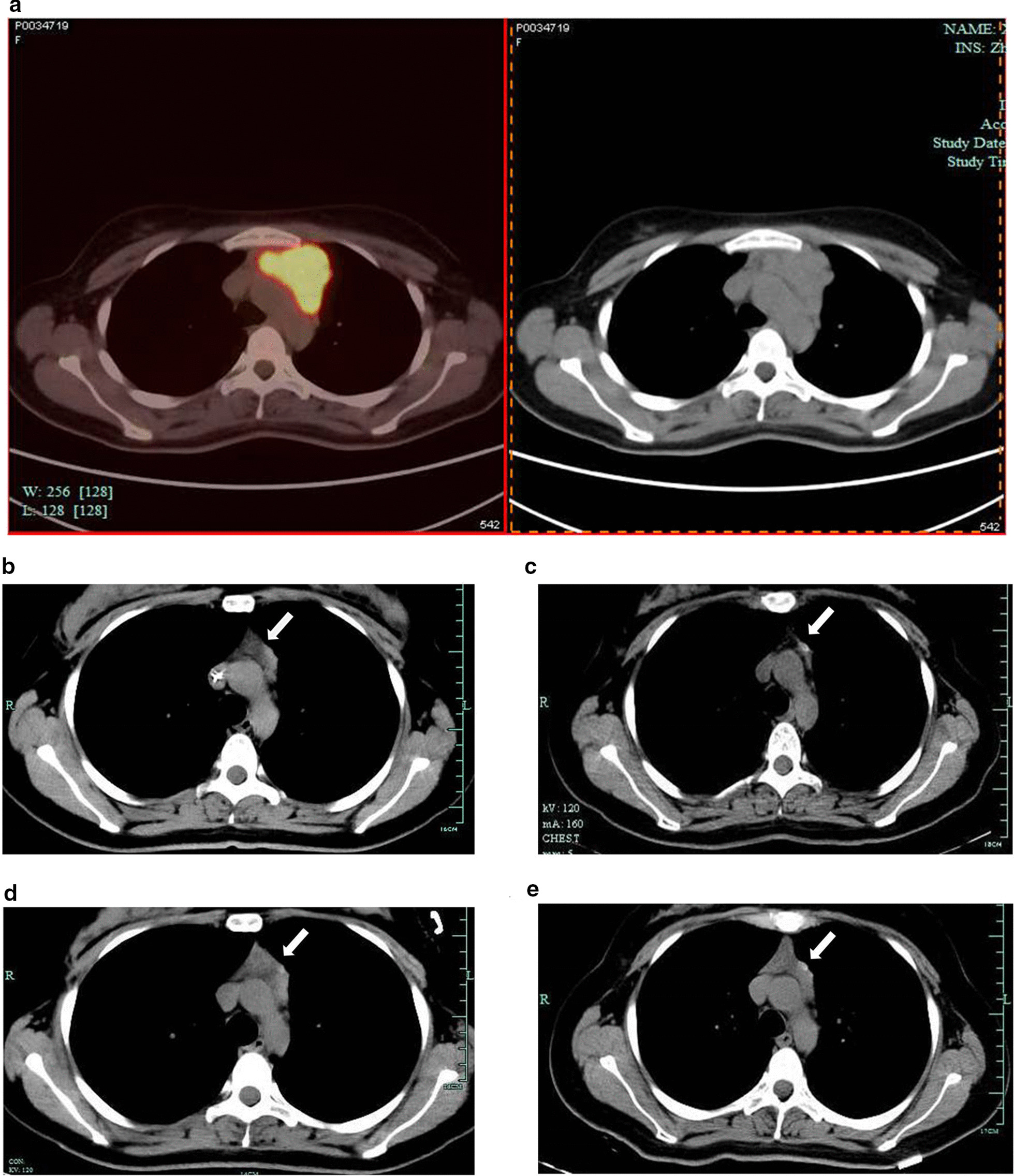
Representative PET/CT or CT images of the superior mediastinal mass in the patient’s chest during treatment. Representative PET/CT images of the anterior mediastinal lesions before treatment, showing multiple lymphadenopathies in the left anterior superior mediastinum and adjacent to the aortic arch, with the maximum SUV of 22 (**a**); After 8 courses of treatment, anterior mediastinal mass could be still seen (**b**); 3 months after ASCT, the anterior mediastinal mass disappeared (**c**); 6 months after ASCT anterior mediastinal mass appeared repeatedly (**d**); 12 months after ASCT, the anterior mediastinal mass observed similar to that 6 months after ASCT (**e**)

Ultrasonography showed multiple enlarged lymph nodes bilaterally around the cervical blood vessels. Cervical lymph node biopsy showed that normal structure of the lymph nodes disappeared and giant cells grew diffusely. Immunohistochemistry of the biopsy showed positive CD20, CD79a, PAX5, LCA, BOB-1, OCT-2, and MUM-1, partially positive Bcl-6 and CD30, 50% positive Bcl-2, 40% positive c-myc, 70% positive Ki-67, and negative CD3, CD5, CD10, CD21, CD15, cyclin D1 and EBER. These results suggested a DLBCL of non-germinal center B-cell origin. Fluorescence in situ hybridization (FISH) showed negative *myc*, *Bcl-2*, and *Bcl-6* rearrangements. Positron emission tomography–computed tomography (PET/CT) showed multiple lymphadenopathies, beneath the right sternocleidomastoid muscle, in the right clavicle, the mediastinal thoracic entrance and the left anterior superior mediastinum, and adjacent to the aortic arch, with the maximum standardized uptake value (SUV) of 22 (Fig. 1a). In addition, focal spleen and bone marrow SUVs were abnormally increased. Considering the above test results, the patient was diagnosed with stage IVA DLBCL of non-germinal center B-cell origin according to the Ann Arbor stage system and with an age-adjusted international prognostic index of 1.

The patient received 4 courses of rituximab-cyclophosphamide, doxorubicin, vincristine, and prednisone chemotherapy (R-CHOP-21) starting on September 24, 2016. The mid-term PET scan after the 4 R-CHOP-21 courses showed that the mediastinal mass adjacent to the aortic arch still existed with the SUV value reduced from the maximum of 22 to 2.6 (hepatic blood pool SUV = 2.2). Given the condition, the patient was subsequently given another 4 courses of R-CHOP-21. At the end of the 6th course of chemotherapy, peripheral blood stem cells were collected following 7.5 μg/kg granulocyte colony stimulating factor (G-CSF) mobilization, and 5.4 × 10^6^/kg hematopoietic stem cells in total were collected. After completion of 8 courses of the chemotherapy, CT scan showed that thickened soft tissue could be seen in the original lesion of the anterior mediastinum (Fig. 1b), while another PET/CT scan showed that the soft tissue inside the left anterior mediastinum and the anterior superior mediastinum was slightly thickened, with slightly increased local density and mild elevation of fludeoxyglucose (FDG) metabolism (SUV = 2.3, hepatic blood pool SUV = 1.9). Tumor activity was therefore could not be ruled out. After discussing the situation with the patient and her family, an autologous hematopoietic stem cell transplantation (ASCT) was performed on July 24, 2017. The treatment regimens included 300 mg/m^2^ carmustine (BCNU) (day − 6), 100 mg/m^2^ etoposide (VP16) (day − 5 to day − 2), 200 mg/m^2^ cytarabine (Ara-c) (day − 5 to day − 2), and 140 mg/m^2^ melphalan (day − 1).

**Fig. 2:**
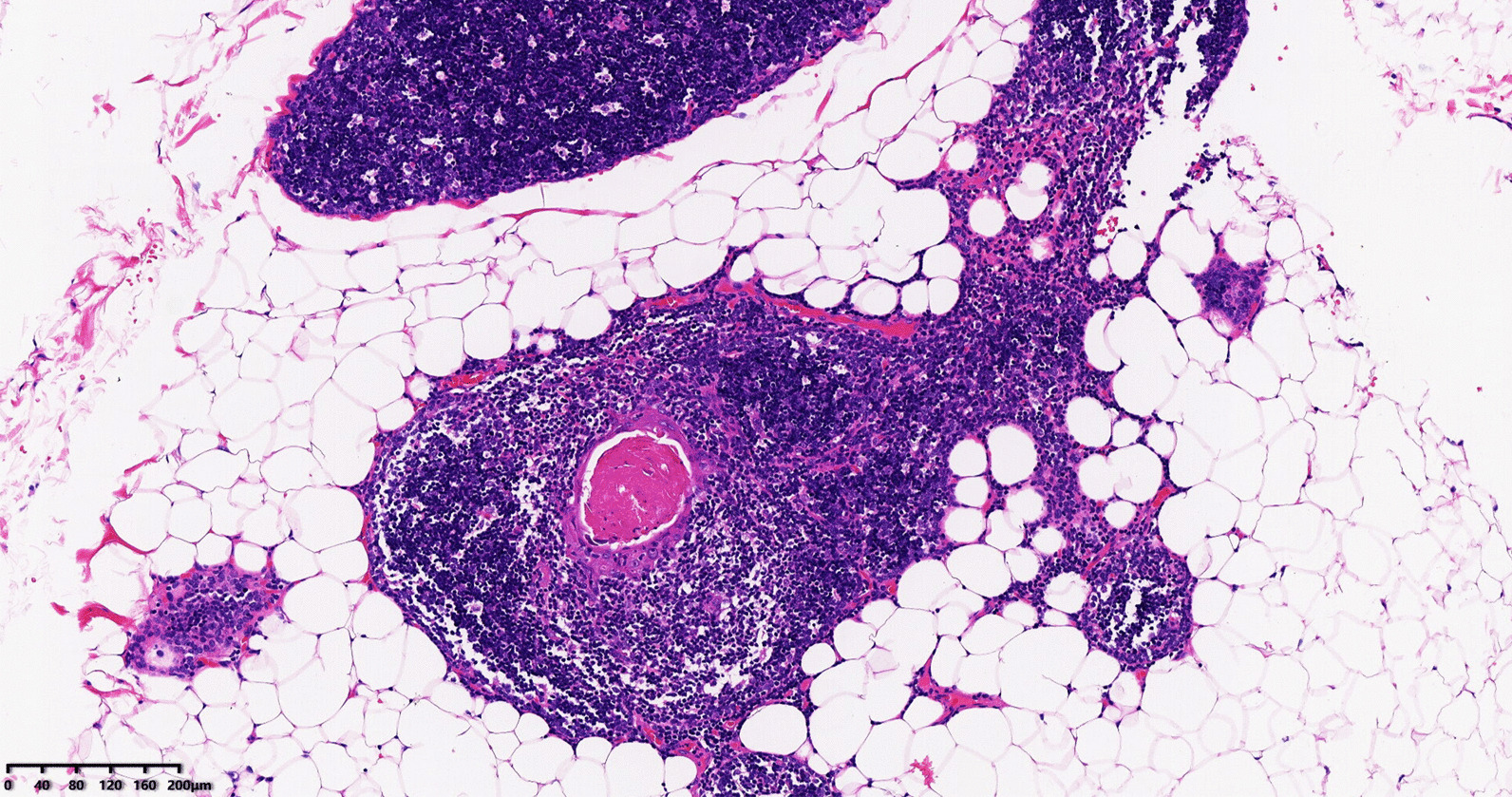
The results of anterior mediastinal biopsy in patients suggested that the thymus tissue. Figure 2 Shows thymus lobule structure, fat hyperplasia and separation, left superior cortex lymphocyte rich (HE staining [h*e*matoxylin–eosin staining] 100)

Further CT scan showed that the thickened soft tissue in the anterior mediastinum almost disappeared 3 months after ASCT (Fig. 1c), while an anterior mediastinal mass was detected again 6 months after ASCT (Fig. 1d), suggesting a potential recurrence of the original lesion. On January 31, 2018, a multi-disciplinary team (MDT), composed of specialists from the Imaging Department (including the specialists from the PET center), Thoracic Surgery Department and the Hematology, was built to make a discussion regarding the condition of the patient. They thought the anterior mediastinal mass was unknown and might be thymus tissue or tumor tissue, and a further observation or a biopsy was required to define. Following another discussion regarding the condition, a video-assisted thoracoscopic surgery was performed for biopsy of the soft tissue of the anterior mediastinum for pathological examination and the result showed thymus tissue (Fig. 2). Final chest CT scan 12 months after ASCT showed an anterior mediastinal mass similar to that on the previous chest CT image (Fig. 1e). The patient has achieved disease-free survival so far. The timeline of the patient is shown in Additional file 1.

**Fig. 3:**
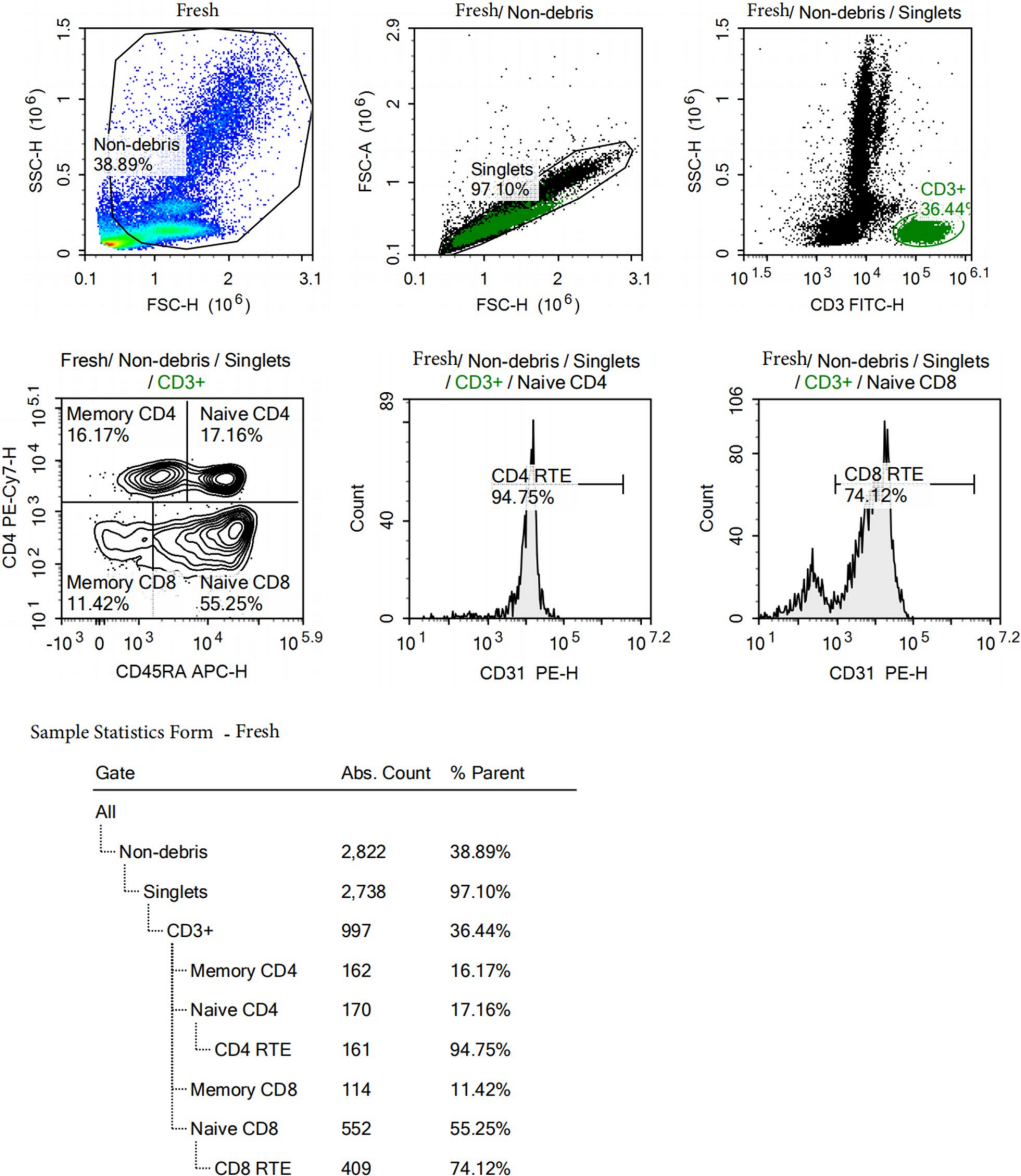
After 6 months of ASCT, the phenotype of peripheral blood T cells was detected by flow cytometry

We analyzed the peripheral blood thymic output of the patient 6 months after ASCT As shown in Fig. 3, the number of total T cells (CD3^+^ T cells) in the peripheral blood was 997/μL, the absolute values of the CD4 RTE, CD8 RTE cells in the peripheral blood were 161/μL and 409/μL respectively.

## Discussion and conclusion

Studies have shown that more than 90% of young patients (≤ 35 years old) with a malignant tumor have premature thymic atrophy due to chemotherapy, but the thymus can regrow after chemotherapy, potentially resulting in reactive thymic hyperplasia or recovery and a diffuse enlargement of the thymus which can be seen on CT (enlarged by 50% or more compared with baseline levels) [8, 9]. As the thymus shrinks with age, additional thymic atrophy caused by chemotherapy may not be obvious in older patients, the ones who also have a limited regenerative capacity [7, 10, 11]. It was reported that in a group of patients aged 18–59 years (median age of 35 years), 30% of patients undergoing chemotherapy had thymic atrophy, and half of them had thymic regeneration during recovery [2]. Also, thymic atrophy can be observed in patients who have ASCT. In view of the abovementioned, the thymus of adults, especially of young adults, evidently preserves its regenerative capacity.

Such thymic hyperplasia is easily mistaken as primary disease progression in clinical practice, and studies on thymic changes after chemotherapy for lymphoma therefore often exclude patients who initially have mediastinal lesions [7, 11]. The young woman in our case report developed anterior superior mediastinal lymphadenopathy which merged into a mass at the time of diagnosis as lymphoma involving lesions, but the thymus was not clearly visible by imaging. The mass significantly shrunk after chemotherapy, while after completion of the therapy, mass appeared repeatedly in the mediastinum on imaging, and residual lymphoma was considered. To eliminate residual lesions, the patient received 8 R-CHOP-21 courses and further ASCT.

In addition to histological verification, there might be other non-invasive methods available to differentiate mediastinal residual lesions from thymic hyperplasia. Studies show that change in thymic volume is often accompanied with change in thymic function.

Thymic output mainly manifests as a recovery of naïve T-lymphocyte number in peripheral blood, which shows that thymic output can be analyzed by detecting T-lymphocyte subsets in peripheral blood using flow cytometry. Sun et al. [7] applied flow cytometry to analyze thymic output in peripheral blood, including single-joint (sj) T-cell receptor excision circles (sjTREC) and CD31 ( +) recent thymic emigrants (RTE), of 54 cases suffering B-cell lymphoma before and after chemotherapy. The results showed that the thymic output cells reduced to its minimal level at the end of chemotherapy, recovered 3 months after the completion of chemotherapy and to a similar or even higher level than the baseline 9 months after the chemotherapy was stopped. Besides, the results also indicated more fast-growing naïve CD4 ( +) T cell and natural regulatory CD4 ( +) T cell subsets in peripheral blood of patients with TH, while similar phenomenon was also noted in tumor patients after chemotherapy reported by Sfikakis et al. [1]

The patient in this case had thymic hyperplasia during the treatment or at the end of chemotherapy which could be found in PET/CT and CT scan. Unfortunately, the possibility was not considered at that time and thymic output cells were not detected. It is reported that patients with an enlarged thymus after chemotherapy have a more rapid recovery of thymic output, suggesting that regrowth of the thymic structure serves as a basis for thymic function renewal [6, 11, 15]. Similarly, examination of thymic output in peripheral blood as a supplementary test can help differentiate thymus tissue from lymphoma in patients with a suspected lesion.

## Supplementary Information

**Additional file 1.** A timeline of the patient.

## Data Availability

All relevant data are provided in the manuscript.

## Abbreviations

TRH: Thymic rebound hyperplasia
PET/CT: Positron emission tomography/computed tomography
SUV: Standardized uptake value
ASCT: Autologous hematopoietic stem cell transplantation
RTE: Recent thymic emigrants

## References

[1] Sfikakis PP (2005). Age-related thymic activity in adults following chemotherapy-induced lymphopenia. Eur J Clin Invest.

[2] Egorov ES (2018). The changing landscape of naïve T cell receptor repertoire with human aging. Front Immunol.

[3] Douek DC (1998). Changes in thymic function with age and during the treatment of HIV infection. Nature.

[4] Clave E (2005). Prognostic value of pretransplantation host thymic function in HLA-identical sibling hematopoietic stem cell transplantation. Blood.

[5] Kumar BV, Connors TJ, Farber DL (2018). Human T cell development, localization, and function throughout life. Immunity.

[6] Hakim FT (2005). Age-dependent insidence, time course, and consequences of thymic renewal in adults. J Clin Invest.

[7] Sun DP (2016). Thymic hyperplasia after chemotherapy in adults with mature B cell lymphoma and its influence on thymic output and CD4(+) T cells repopulation. Oncoimmunology.

[8] Choyke PL, Zeman RK, Gootenberg JE (1987). Thymic atrophy and regrowth in response to chemotherapy: CT evaluation. AJR Am J Roentgenol.

[9] Zhen Z, Sun X, Xia Y (2010). Clinical analysis of thymic regrowth following chemotherapy in children and adolescents with malignant lymphoma. Jpn J Clin Oncol.

[10] Hara M, McAdams HP, Vredenburgh JJ (1999). Thymic hyperplasia after high-dose chemotherapy and autologous stem cell transplantation: incidence and significance in patients with breast cancer. AJR Am J Roentgenol.

[11] Sun DP, Wang L, Ding CY (2016). Investigating factors associated with thymic regeneration after chemotherapy in patients with lymphoma. Front Immunol.

[12] Hakim FT, Gress RE (2002). Reconstitution of thymic function after stem cell transplantation in humans. Curr Opin Hematol.

[13] Kohler S, Thiel A (2009). Life after the thymus: CD31+ and CD31- human naïve CD4+ T-cell subsets. Blood.

[14] Storek J (2002). Factors influence T-lymphopoiesis after allogeneic hematopoietic cell transplantation. Transplantation.

[15] Boehm T, Swann JB (2013). Thymus involution and regeneration: two sides of the same coin?. Nat Rev Immunol.

