# Supplementary material for: Thymic rebound hyperplasia post-chemotherapy mistaken as disease progression in a patient with lymphoma involving mediastinum: a case report and reflection
Source: BMC Surg. 2021 Jan 14;21:38. doi: 10.1186/s12893-021-01048-y (PMC7809830; doi:10.1186/s12893-021-01048-y)
Supplement: Supplementary file 1 — Additional file 1. A timeline of the patient. [file 12893_2021_1048_MOESM1_ESM.docx]

A timeline of the patient

2016-9-13 Cervical mass was discovered.

2016-9-20 The patient was diagnosed as DLBCL by Cervical lymph node biopsy, and PET scan showed multiple lymphadenopathies in the left anterior superior mediastinum and adjacent to the aortic arch with the maximum SUV of 22.

2016-9-24 The patient received R-CHOP-21 regimen, and PET/CT after 4 courses showed few active mediastinal mass adjacent to the aortic arch, suggesting partial remission.

2017-6-2 PET/CT after completion of 8 courses of chemotherapy showed an anterior mediastinal mass with mild elevation of FDG metabolism higher than that of the hepatic blood pool.

2017-7-24 ASCT

2017-10-25 CT after 3 months of ASCT showed anterior mediastinal mass disappeared.

2018-1-24 CT after 6 months of ASCT showed anterior mediastinal mass appeared repeatedly and enlarged.

2018-2-27 The patient received thoracoscopic surgery and pathological examination showed the anterior mediastinal mass as thymus tissue.

2018-1-31 MDT was performed and the patient's condition was repeatedly discussed with the patient and her family.
